# Young Adult Patient and Caregiver Perspectives on Transition Readiness in an Inflammatory Bowel Disease Clinic

**DOI:** 10.1093/crocol/otae044

**Published:** 2024-07-26

**Authors:** Thomas M Strobel, Nikita A Narayani, Maribeth R Nicholson, Diana C Riera, Tanner G Rakos, Nisa P Fulton, Jordan A Trotter-Busing, Sara N Horst, Robin L Dalal, Baldeep S Pabla, Elizabeth A Scoville, David A Schwartz, Dawn B Beaulieu

**Affiliations:** Division of Gastroenterology, Hepatology, and Nutrition, Department of Medicine, Vanderbilt University Medical Center, Nashville, TN, USA; Division of Gastroenterology, Hepatology, and Nutrition, Department of Medicine, Vanderbilt University Medical Center, Nashville, TN, USA; D. Brent Polk Division of Gastroenterology, Hepatology and Nutrition, Department of Pediatrics, Vanderbilt University Medical Center, Nashville, TN, USA; D. Brent Polk Division of Gastroenterology, Hepatology and Nutrition, Department of Pediatrics, Vanderbilt University Medical Center, Nashville, TN, USA; Division of Gastroenterology, Hepatology, and Nutrition, Department of Medicine, Vanderbilt University Medical Center, Nashville, TN, USA; Division of Gastroenterology, Hepatology, and Nutrition, Department of Medicine, Vanderbilt University Medical Center, Nashville, TN, USA; D. Brent Polk Division of Gastroenterology, Hepatology and Nutrition, Department of Pediatrics, Vanderbilt University Medical Center, Nashville, TN, USA; Division of Gastroenterology, Hepatology, and Nutrition, Department of Medicine, Vanderbilt University Medical Center, Nashville, TN, USA; Division of Gastroenterology, Hepatology, and Nutrition, Department of Medicine, Vanderbilt University Medical Center, Nashville, TN, USA; Division of Gastroenterology, Hepatology, and Nutrition, Department of Medicine, Vanderbilt University Medical Center, Nashville, TN, USA; Division of Gastroenterology, Hepatology, and Nutrition, Department of Medicine, Vanderbilt University Medical Center, Nashville, TN, USA; Division of Gastroenterology, Hepatology, and Nutrition, Department of Medicine, Vanderbilt University Medical Center, Nashville, TN, USA; Division of Gastroenterology, Hepatology, and Nutrition, Department of Medicine, Vanderbilt University Medical Center, Nashville, TN, USA

**Keywords:** inflammatory bowel disease, transition of care, adolescents, young adults, caregivers

## Abstract

**Background:**

When it comes to readiness to transition to an adult subspecialty clinic, perspectives between patients with inflammatory bowel disease (IBD) and their caregivers may differ and influence the ability to successfully transition. Patients with IBD have been shown to suffer from poor transfers of care. There is a need to more efficiently and accurately assess transition readiness to improve the transfer process.

**Methods:**

Patients transferring to an adult subspecialty clinic and their caregivers were each administered the Transition Readiness Assessment Questionnaire and IBD Self-Efficacy Scale—Adolescent. Differences between patient and caregiver responses and agreement among each dyad were tested.

**Results:**

There were 29 dyads of patients and caregivers who enrolled. There was no difference between patient and caregiver total scores. The average level of agreement between patients and caregivers was 78%. There was no association between patient response and their age, gender, ethnicity, age at time of transfer, age at diagnosis, or number of emergency room visits in the prior year.

**Conclusions:**

Patient-reported readiness to transition to adult care was confirmed by their caregivers using validated readiness assessment tools. As transition clinics must focus on high-yield interventions, a readiness survey of young adult patients without a survey of their caregivers may be adequate. However, as experts in each patient’s journey, caregivers may be utilized when setting goals and priorities for a transition readiness program. The surveys used in this study can be used broadly to aid subspecialty clinics that are trying to improve the transition process.

## Graphical Abstract

**Graphical Abstract:**
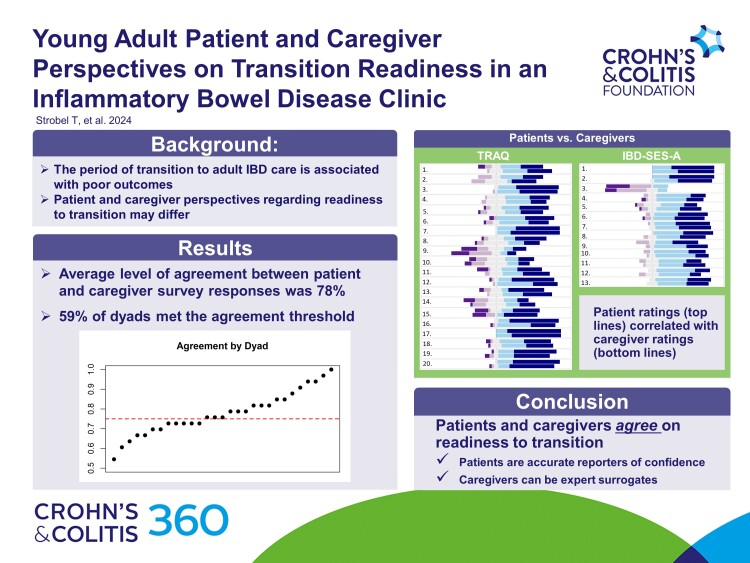

## Introduction

A transition of care is the process of transferring care from a pediatric to an adult provider. Children with chronic, complex diseases are vulnerable to poor outcomes during the transition period. This includes patients with inflammatory bowel disease (IBD) who, during the transition period, are at risk for disease progression, healthcare overutilization, and psychological distress.^1–5^ These experiences negatively impact school performance and peer relationships.^6,7^ It is challenging to improve the transition process when there is no widely accepted standard for success. Instead, experts focus on certain skills to prepare families for the transition. These skills, which relate to patient autonomy and self-efficacy, are commonly assessed by clinics when determining readiness to transition.

Improving the transition process starts with identifying the important stakeholders, which include the patients, the adult and pediatric providers, the caregivers, and the multidisciplinary support staff.^4^ Beyond this, we need to recognize each person’s role within the team. Without clear roles, transition programs risk implementing costly and inefficient interventions in a setting where time and resources are already limited.

This study focuses on the role of the caregiver in the IBD transition. In pediatric gastroenterology, providers often utilize caregivers as patient surrogates during the medical interview; however, it is not clear what aspects of the caregiver’s perspective are useful during the transition interview, or if caregiver perspectives accurately represent those of the young adult patients. Previous studies that attempted to compare the perceptions of young adult patients with their caregivers in regard to symptoms, self-efficacy, and quality of life found mixed results.^2,8–11^ Some have found that caregivers have a tendency to overestimate the patient’s symptoms and underestimate their quality of life.^9,12^ Other studies have shown that caregivers agree with patients on quality of life.^12,13^ The inconsistency of these findings highlights the lack of a defined role for caregiver assessments.

This study aims to better understand the caregiver’s role by comparing patient and caregiver responses to 2 widely used transition readiness-specific questionnaires that are validated in the young adult IBD population—the Transition Readiness Assessment Questionnaire (TRAQ) and the IBD Self-Efficacy Scale—Adolescent (IBD-SES-A).^14,15^ The findings of this study will help clinicians to better utilize caregivers during the transition process.

## Materials and Methods

### Patients and Procedures

From September 2022 to April 2023, patients diagnosed with IBD, based on provider assessment, who established care at a single adult IBD specialty clinic and had seen a pediatric gastroenterologist within 3 years prior to enrollment were assessed for eligibility. Patients were administered the TRAQ and the IBD-SES-A either in person before their appointment or via an online survey tool.^14,15^ Caregivers were also asked to complete the same 2 questionnaires and answer on behalf of the patient. Demographic information was self-reported when appropriate or collected via chart review. Number of emergency room visits in the previous year was collected via chart review. All data were stored in a REDCap database.^16^

### Measures

The TRAQ is a measure of transition readiness for adolescents and young adults. The questions elicit the participant’s confidence in their ability to manage their medications, keep appointments, track their health issues, talk with providers, and manage daily activities. The assessment includes 20 questions and asks the individual to rate their confidence to perform certain skills from “No, I do not know how” being the lowest confidence to “Yes I always do this when I need to” being the highest confidence. Ratings were graded on a scale of 1 to 5 with a higher score indicating higher confidence.^14,17^The TRAQ is divided into 5 domains: “Managing Medications” includes questions 1–4, “Appointment Keeping” includes 5–11, “Tracking Health Issues” includes 12–15, “Talking with Providers” includes 16-, and “Managing Daily Activities” includes 18–20.

The IBD Self-efficacy Scale—Adolescent is a 13-question survey that elicits the patient’s level of confidence in managing various aspects of their daily life as they pertain to IBD. Each item is scored on a scale of 1 (completely disagree) to 5 (completely agree), and higher scores reflect greater self-efficacy (ie, “perception of one’s ability to engage in skills required to master a new challenge despite obstacles”).^15^

Lastly, each individual patient’s situation at the time of transfer was categorized by the study investigator (***) into 1 of the 4 situations: Typical, nonengaged, crisis, or special needs via criteria proposed by Philpot et al.^18^ A “typical” situation is a young adult ≥18 years of age, independent, with disease in remission and ready to transfer care. The “non-engaged” type is a patient not involved in the clinic with few or no recent clinic visits. The “crisis” type is defined as uncontrollable disease necessitating a transfer for surgery or newer medications, pregnancy, conflict with the pediatrician, or substance use. The “special needs” transfer is a patient with an intellectual disability or comorbidity that causes a challenge to an adult practitioner who has less experience in this type of care.

### Outcomes

The primary aim was to measure the relationship between patient and caregiver responses to the TRAQ and IBD-SES-A. Each question was individually analyzed, and scores were combined into composite TRAQ domain scores and total scores for both questionnaires.

The secondary aims were to measure the degree of agreement between each dyad, determine topics with the greatest and least confidence among the cohort, and test associations between patient scores and various demographic factors including age, sex, ethnicity, diagnosis, age at diagnosis, age at transfer of care, emergency department (ED) visits, and transfer type.

### Statistical Analysis

Statistical analysis was performed using R version 4.1.2 (https://cran.r-project.org/, accessed July 05, 2023). Nominal variables are described as count *n* (% frequency). Continuous variables are reported as mean (SD) or median [MIN, MAX] if a distribution is not normal. Normality was tested using the Shapiro–Wilk test. The Wilcoxon signed-rank test was used to test for differences between total and individual patient and caregiver scores. Prior to analysis, a power setting of 0.8 with a significance level of 0.05 and an estimated effect size of 0.72 were used to determine an enrollment goal of 15. Total scores for each survey are reported as raw numbers. The TRAQ domains are reported as a percentage of the total possible points per domain.

Agreement was defined as the percentage of congruent, dichotomized responses (“yes” [4–5] or “no” [1–3] for TRAQ and “agree” [4–5] or not [1–3] for IBD-SES-A), where 1.0 represents perfect agreement. The cutoff for agreement was set at 0.75—a benchmark which has been used in similar methodologies.^8^

A 2-sample *t*-test was used to test for the influence of sex, ethnicity, and IBD diagnosis on total scores (TRAQ and IBD-SES-A). Ordinal logistic regression was used to test for a relationship between total scores and age, age at diagnosis, age at time of transfer, and ED visits.

## Ethical Considerations

This study was reviewed and approved by the *** Institutional Review Board. It is an original contribution and is not under current consideration for publication nor has it been previously published elsewhere. It was presented as a poster at the American College of Gastroenterology Annual Scientific Meeting in 2023.

## Results

### Demographics

There were 42 young adult patients transferring care to a single adult IBD specialty clinic who were screened for enrollment. Four patients were deemed ineligible as they had not seen a pediatric gastroenterologist within 3 years prior to transfer. Of the 38 eligible patients, 33 enrolled and completed both surveys. Of these, 29 patients also had a caregiver who completed both surveys individually, resulting in 29 dyads. Table 1 summarizes the demographic information for the 33 patients who completed both surveys. Patient ages ranged from 18 to 22 years. Five of the thirty-three patients had one emergency room visit in the previous year, 1 had 2 visits, one had 4 visits, one had 6 visits and the rest had 0 visits.

**Table 1.** Demographics of patients with inflammatory bowel disease who completed the surveys.

	Overall (*N* = 33)
*Age at the time of survey (years)*	
Mean (SD)	19.1 (1.10)
*Sex*	
Female	15 (45.5%)
Male	16 (48.5%)
n/a	2 (6.1%)
*Ethnicity*	
Hispanic/Latino	2 (6.1%)
Non-Hispanic/Latino	31 (93.3%)
*IBD disease subtype*	
Crohn’s disease	20 (60.6%)
Ulcerative colitis	11 (33.3%)
Indeterminate colitis	2 (6.1%)
*Age at diagnosis (years)*	
Mean (SD)	13.2 (3.29)
*Age at time of transfer (years)*	
Mean (SD)	18.7 (0.977)
*Transition type*	
Typical	32 (97.0%)
Nonengaged	0 (0%)
Crisis transfer	1 (3.0%)
Special needs	0 (0%)

### Patients Versus Caregivers

**Figure 1.**
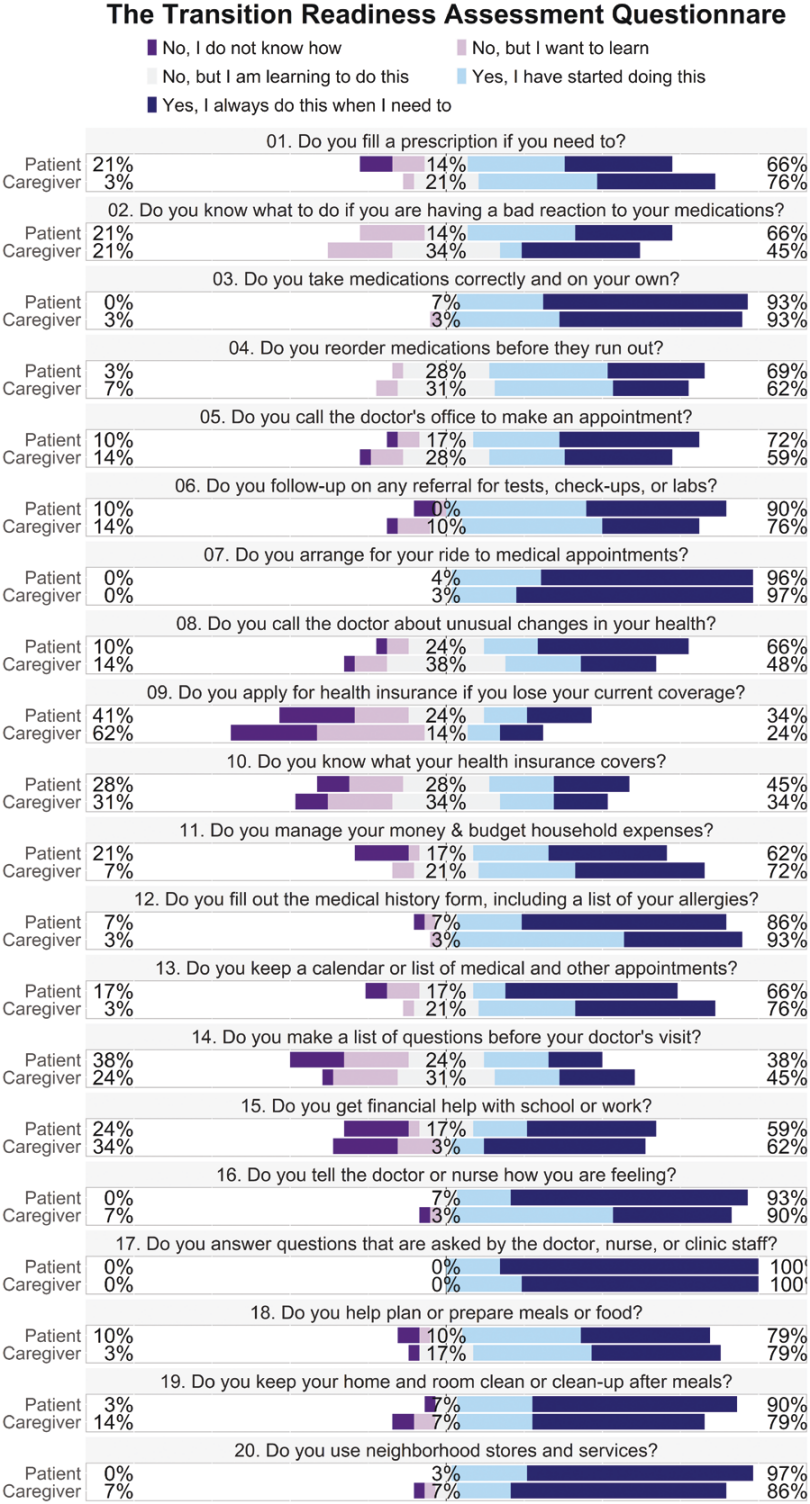
Transition Readiness Assessment Questionnaire score composites. Percentages at far left and right reflect combined “No” or “Yes” answers. Percentages for neutral answers are in the middle.

**Figure 2.**
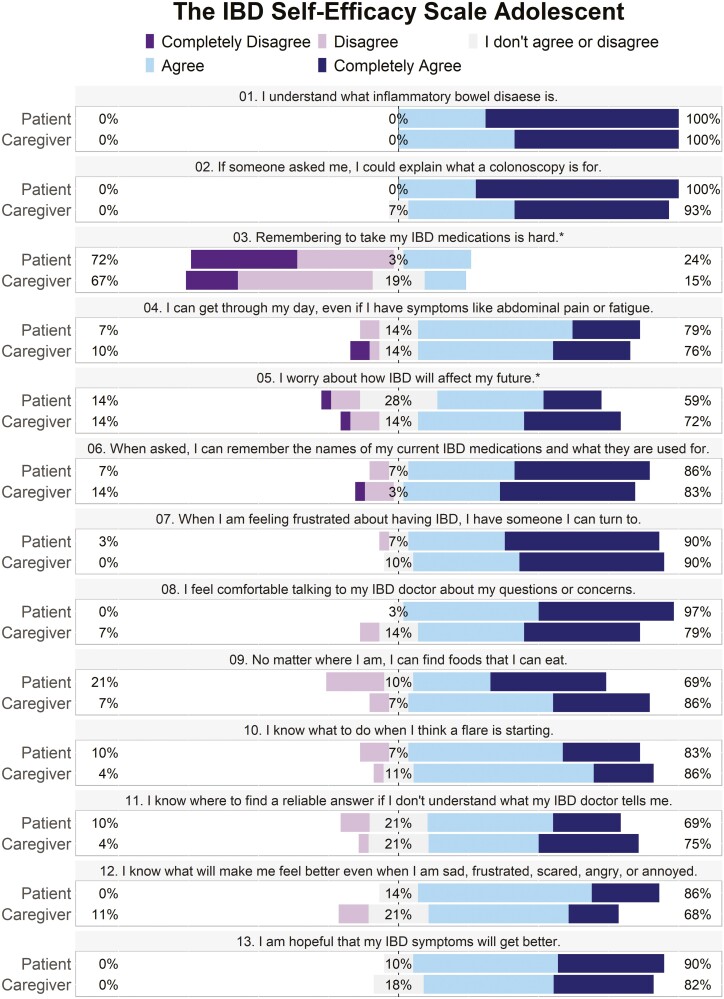
Inflammatory bowel disease-SES-A score composites. Percentages at far left and right reflect combined “Disagree” or “Agree” answers. Percentages for neutral answers are in the middle. *Items 3 and 5 are reverse scored.

**Figure 3.**
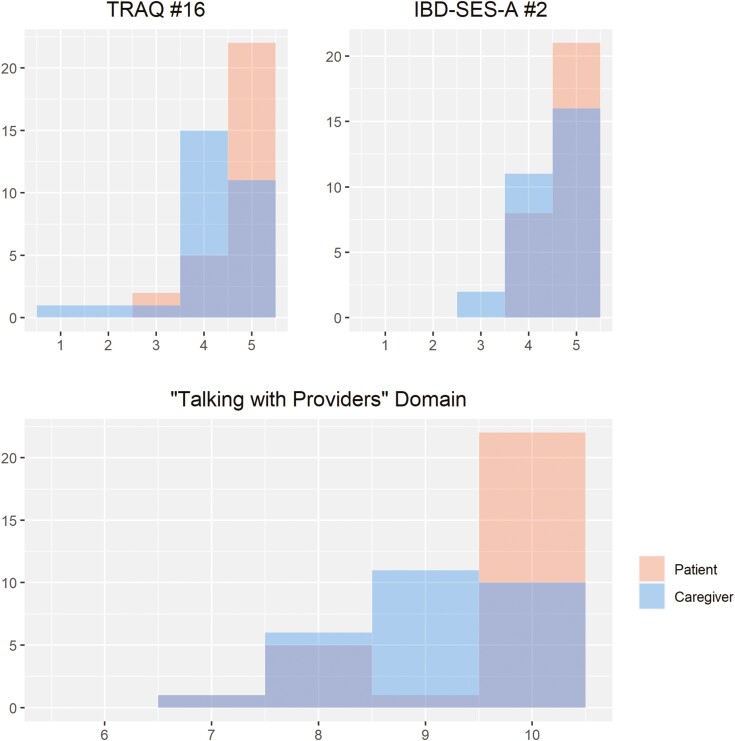
Frequencies of scores for patients and caregivers on the Transition Readiness Assessment Questionnaire (TRAQ) #16, inflammatory bowel disease-SES-A #2, and TRAQ Domain: “Talking with Providers.”

**Figure 4.**
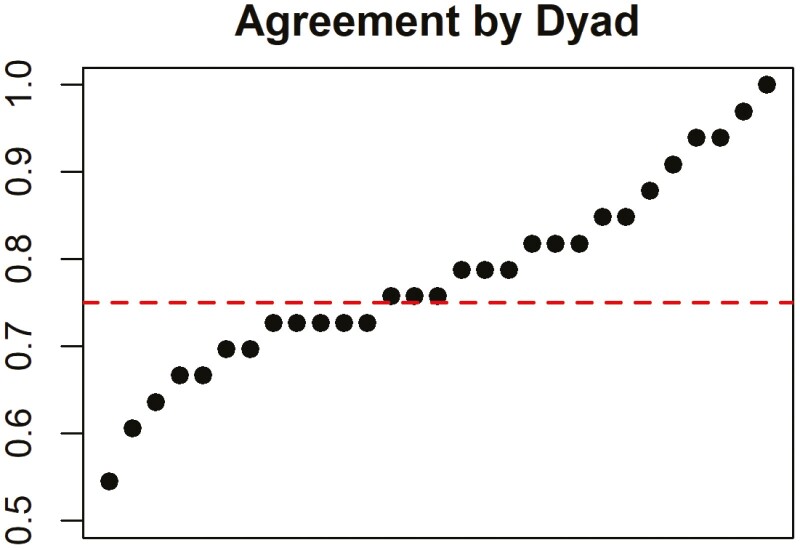
Degree of agreement for the cohort of dyads. Each point represents a dyad. The line represents the threshold of acceptable agreement (ie, 0.75).

The results of the TRAQ and IBD-SES-A are displayed in Figures 1 and 2, respectively. For the patient group, the average score on the TRAQ was 80.3 (SD 11.6) and on the IBD-SES-A was 52.4 (SD 5.29). For caregivers, the average score on the TRAQ was 78.7 (SD 13.5) and on the IBD-SES-A was 51.9 (SD 5.20). There was no statistically significant difference between patient and caregiver total scores for the TRAQ (*P* = .626) or IBD-SES-A (*P* = .746). There were 2 individual questions with statistically significant differences: question number 16 on the TRAQ, “telling the doctor or nurse how you are feeling,” (5.0 [3.0, 5.0] vs. 4.0 [1.0, 5.0], *P* = .001) and question number 2 on the IBD-SES-A, “I could explain what a colonoscopy is for,” (5.0 [4.0, 5.0] vs. 5.0 [3.0, 5.0], *P* = .039) where in both instances the patients reported higher confidence than their caregivers (Figure 3). Patients also reported higher confidence in the “Talking with Providers” TRAQ domain compared to caregivers (10.0 [7.0, 10] vs. 9.0 [5.0,10], *P* = .027; Table 2). Average level of agreement among the dyads was 0.78 (SD 0.11) and ranged from 0.55 to 1.00. Of the 29 dyads, 17 (59%) met the agreement threshold of 0.75, while 12 did not (Figure 4).

**Table 2.** Total Transition Readiness Assessment Questionnaire scores by domain.

	Patient (*N* = 29)	Caregiver (*N *= 29)	*P*-value
Managing medications	16 [10, 20]	16 [8.0, 20]	.698
Appointment keeping	28 [12, 35]	26 [13, 35]	.389
Tracking health issues	16 [7.0, 20]	16 [9.0, 20]	.499
Talking with providers	10 [7.0, 10]	9.0 [5.0, 10]	.027
Managing daily activities	14 [9.0, 15]	14 [5.0, 15]	.302

### Transition Readiness and Self-Efficacy

Of the 33 patients who completed both surveys, questions with the highest confidence included question 1 of the IBD-SES-A, “I understand what [IBD] is,”(4.7, SD 0.45), question 2 of the IBD-SES-A, “…I could explain what a colonoscopy is for,” (4.7, SD 0.45), question 16 of the TRAQ “…tell the doctor how you are feeling,” (4.6, SD 0.57), question 17 of the TRAQ, “…answer questions that are asked by the doctor…,” (4.8, SD 0.36), and question 20 of the TRAQ, “use neighborhood stores and services,” (4.7, SD 0.52). Questions with the lowest confidence included question 5 of the IBD-SES-A, “I worry about how IBD will affect my future,” (2.5, SD 1.1), question 9 of the IBD-SES-A, “…apply for health insurance when you lose coverage,” (3.0, SD 1.5), and question 14 of the TRAQ, “make a list of questions…” (2.9, SD 1.3). Domains ranked by most to least confidence were: “Talking with Providers” (0.96, SD 0.087), “Managing Daily Activities” (0.88, SD 0.12), “Managing Medications” (0.81, SD 0.14), “Appointment Keeping” (0.77, SD 0.18), and “Tracking Health Issues” (0.73, SD 0.19). There was no significant association between age, sex, ethnicity, age at the time of transfer, age at diagnosis, transition type, IBD diagnosis, or ED visits on total TRAQ or IBD-SES-A scores.

## Discussion

The transition of care for patients with IBD from pediatric to adult providers is a significant challenge, and inadequate transitions have been associated with a host of negative outcomes. Improving the transition is an area of need, and this starts with clarifying each stakeholder’s role. Through the careful surveying of patients with IBD who recently transitioned to an adult gastroenterology clinic and their caregivers, this study showed significant agreement between patients and caregivers in terms of transition readiness based on both TRAQ and IBD-SES-A scores. Therefore, patient-reported confidence in key aspects of transition readiness was confirmed by their caregivers.

Prior studies on transition readiness have shown widely varying levels of agreement between patients and caregivers, ranging from 22% to 84%, with a tendency for older subjects to have higher agreement.^8,9,12^ Compared to these previous studies, the current study was comprised of an older population, and 59% of the dyads met the agreement threshold. It is possible that older age and developmental maturity led to higher agreement. This also may be a reason why there was a minimal overestimation of self-efficacy by the caregiver group, which is in contrast to a previous study with a younger cohort.^19^ The current study demonstrates that while it may not be necessary to routinely include caregivers on readiness surveys with overall survey agreement, they should be allowed some feedback on transition readiness even as the patient ages and matures.

The patients scored below average for their age in each of the TRAQ domains when compared to the reference scores in the validation study published by van Gaalen et al.^14^ In that study, poorer performance was linked to fewer transition visits, and nearly half of the cohort had received multiple readiness assessments. While the number of prior transition assessments per patient was not collected as part of this study, at our institution, patients routinely have a single transition visit where they receive a depression screen (Patient Health Questionnaire-9) and review the NASPGHAN transition checklist.^20^ Overall, fewer transition visits and assessments per patient might explain the lower TRAQ scores in the study population, and it may highlight a key difference in transition practices among individual clinics and internationally.

The topics that patients expressed the lowest confidence in included knowing how IBD would affect their future, and how to acquire health insurance if they were to lose coverage. These sentiments were consistent with findings of a previous study in this population, where disease uncertainty and lack of control negatively impacted quality of life.^2^ Of all the topics assessed, these may be the most pervasive in young adulthood and expectations may need to be discussed more thoroughly in the transition process. Insurance coverage questions might best be addressed via standardized materials or a social worker.

There were a few instances where caregivers underestimated patient confidence (“telling the doctor how you are feeling” and “…explain what a colonoscopy is for”). It is possible that this is a more accurate reflection of the patient’s true skill level or that the caregivers are under-reporters of the patient’s skill level. A longitudinal evaluation of transition success would be beneficial to help understand these differences. Education on these topics earlier in the transition process could be beneficial, including educating children about the colonoscopy procedure and teaching them useful skills to participate in the clinic visit (ie, bringing a list of questions for the doctor).

Experts in transition of care recommend starting the process as early as 12 or 14 years of age, and the principles of transition should be included in each clinic visit.^21^ It is recommended that families be given the written transition policy at the start to set expectations and allow families to begin to practice important skills at home. While some families may need more time and education to prepare for transition, frequent readiness assessments such as the 20-question TRAQ, which can be administered during the intake process, can help to identify areas of proficiency that do not necessarily need to be addressed at every visit. Additionally, this allows programs to identify common areas of deficiency to implement broader interventions for all patients. Based on the results of the current study and consistent with most recommendations, we would encourage providers to interview patients alone in the room as they would be expected to do in an adult clinic. It may be beneficial to elicit caregiver perspectives upon completion of the initial interview, particularly if the caregiver expresses readiness concerns.

There were some limitations to this study. First, the study population was likely not a full representation of the IBD population. Racial, ethnic, and gender-diverse persons with IBD are less reported in the literature and have less access to quality healthcare.^22,23^ Future studies should aim to enroll a cohort more reflective of all patients within the population to provide equitable care. Second, there are limitations to using Likert scales. A central tendency bias, or avoidance of extreme responses, and social desirability bias, or attempting to portray oneself as more favorable, can confound the data. Recent studies have been successful in defining benchmarks for the TRAQ in patients with IBD.^14^ The same should be done for the IBD-SES-A to ensure the data is protected from confounding variables. Additionally, while the few who completed online surveys received them via their individual emails, we could not verify that the patients did not let caregivers answer for them, which would influence agreement. Lastly, while the population was from a subspecialty clinic, we know that many patients came from an institution with a pediatric clinic with a known transition program. Therefore, this study population may not be generalizable to centers without a formal transition program, and this may also influence the patient and caregiver agreement. Continued efforts to define markers of a successful transition for IBD patients are needed to help providers, patients, and caregivers target areas that are most critical. The larger role of TRAQ and the IBD-SES-A tools in relation to a successful transition warrants further study.

## Conclusion

The process of improving the transition from pediatric to adult IBD care starts with identifying and clarifying each stakeholder’s role. In this study, patient confidence was confirmed by their caregivers using validated transition readiness metrics. In an effort to support their transition to adult care, we suggest focusing on patient perspectives when assessing transition readiness. As experts with special experience, caregivers may serve as additional reporters during the assessment period. A standardized readiness assessment tool, such as the TRAQ or IBD-SES-A, can help subspecialty clinics assess their young adult patients who are preparing to transition to adult-based care models.

## Data Availability

Data pertaining to this study will be made available to individuals upon request to the corresponding author.
